# A Community-Based, Technology-Supported Health Service for Detecting and Preventing Frailty among Older Adults: A Participatory Design Development Process

**DOI:** 10.1155/2015/216084

**Published:** 2015-08-05

**Authors:** Lex van Velsen, Maddalena Illario, Stephanie Jansen-Kosterink, Catherine Crola, Carolina Di Somma, Annamaria Colao, Miriam Vollenbroek-Hutten

**Affiliations:** ^1^Roessingh Research and Development, Telemedicine Cluster, P.O. Box 310, 7500 AH Enschede, Netherlands; ^2^University of Twente, Biomedical Signals and Systems Group, P.O. Box 217, 7500 AE Enschede, Netherlands; ^3^Federico II University, Department of Translational Medical Sciences, Largo S. Marcellino 10, 80138 Naples, Italy; ^4^Federico II University Hospital, Research & Development, Largo S. Marcellino 10, 80138 Naples, Italy; ^5^Federico II University Hospital, Department of Clinical Medicine and Surgery, Largo S. Marcellino 10, 80138 Naples, Italy; ^6^Federico II University Hospital, Department of Gastroenterology, Endocrinology and Surgery, Largo S. Marcellino 10, 80138 Naples, Italy

## Abstract

Frailty is a multifaceted condition that affects many older adults and marks decline on areas such as cognition, physical condition, and nutritional status. Frail individuals are at increased risk for the development of disability, dementia, and falls. There are hardly any health services that enable the identification of prefrail individuals and that focus on prevention of further functional decline. In this paper, we discuss the development of a community-based, technology-supported health service for detecting prefrailty and preventing frailty and further functional decline via participatory design with a wide range of stakeholders. The result is an innovative service model in which an online platform supports the integration of traditional services with novel, Information Communication Technology supported tools. This service is capable of supporting the different phases of screening and offers training services, by also integrating them with community-based services. The service model can be used as a basis for developing similar services within a wide range of healthcare systems. We present the service model, the general functioning of the technology platform, and the different ways in which screening for and prevention of frailty has been localized. Finally, we reflect on the added value of participatory design for creating such health services.

## 1. Introduction

Aging can be considered a success story for health policies in the modern world. And while living longer is a beautiful thing, it also comes with downsides, such as frailty. Frail older adults are those “who are at increased risk for future poor clinical outcomes, such as development of disability, dementia, falls, hospitalization, institutionalization, or increased mortality” [1]. As such, frailty is a state that consists of many dimensions. Physical and cognitive decline, as well as malnutrition, have been identified as the major dimensions of frailty among older adults [2]. Becoming frail is something that happens gradually. A person starts out robust (or healthy), becomes prefrail (a stage in which components of frailty become manifest), and can then evolve to become frail (which can be observed clinically easily) [3]. In ten European countries, the percentage of prefrail people among community-dwelling older adults have been found to range from 30.4% to 44.9%, and the percentage of frail people among this group ranged from 1.3% to 5.9% [4].

Determining the level of frailty among older adults and offering them interventions to train their health have been found to help them live at home independently for a longer amount of time and to reduce the rate of falls [5] or helps them to maintain their functional capacity [6]. However, current identification methods for frailty and prefrailty among older adults are resource-intensive and more efficient (but equally reliable) alternatives need to be developed [7]. This need aligns with the great challenges that society has to deal with in organizing its healthcare. Demand is rising due to a population that includes increasingly more older adults that have to be supported by increasingly fewer young people; older adults should be able to function in society independently and without drawing too much on society's resources; increasingly high standards of living lead to higher expectations with regard to the quality of care while budgets are being tightened [8]. Two types of healthcare services have been identified as possible solutions to these problems: community-based services and eHealth [9, 10]. A community-based healthcare service is a form of care that is provided out of medical institutions within a patient's community. This does not mean that healthcare professionals (e.g., a general practitioner) are not involved. Rather, the majority of the service is provided at a location in a patient's neighborhood that is not care-related (e.g., the community house) and involves a collaboration of healthcare professionals, local organizations, and volunteers. For the context of care for older persons, such services are meant to help older people live independently in their own house while maintaining their quality of life [11]. Community-based services can be a method to decrease the pressure on the healthcare system by moving care from medical institutions towards local community services. Collaborations between primary care, volunteers, and community services have been shown, among other things, to be able to predict poor nutrition among older adults [12], to prevent hospitalizations and major disabilities among chronically ill older adults [13], and to prevent the number of falls among older adults with a history of falling or having concerns about falling [14].

eHealth (or “health services and information delivered or enhanced through the internet and related technologies” [15]) has been proposed as a solution for overcoming the aforementioned challenges and is associated with great promises, such as improved access to health information, greater quality of care, and higher adoption of healthy behavior [16]. By means of eHealth services, patients can be empowered to take care of themselves, assisted by technology. This way, the burden on the healthcare system decreases, while patients gain control.

A community-based, technology-supported service model for screening for frailty among older adults and training their health can be a cost-effective alternative for the clinically based services that are currently available. By combining both approaches, care is moved away from care institutions, while it allows older adults to choose which kind of service they prefer (face-to-face in the community or via online self-service technology). But creating such a service means that new working procedures need to be devised. Therefore, involvement from stakeholders and potential end-users is extremely important [17, 18]. In this paper, we discuss the development of a community-based, technology-supported service model that aims to detect and prevent frailty among older adults, by means of a participatory design approach.

## 2. Materials and Methods

### 2.1. Participatory Design

We approached the design of the service model by means of participatory design. Participatory design is a design approach that advocates the inclusion of all stakeholders (including potential end-users and/or their representatives) during design activities in order to come to a design solution that aligns with all stakeholders' and end-users' needs and context [19, 20]. We conducted a series of workshops in which stakeholders and end-user (representatives) were gathered with multiple goals. First, we wanted to create awareness of the upcoming services. Second, we gathered direct input from the people that we anticipated to be actually working with the service or were going to be affected by it for service design. And third, as the workshop participants, for a large part, were also going to be the persons working with in the service, providing them input would them a sense of responsibility and ownership that, in turn, leads to higher acceptance [21].

### 2.2. Project Context

The design of the service model was part of the European FP7 project PERSSILAA (which stands for Personalised Information Communication Technology (ICT) Supported Services for Independent Living and Active Ageing). This project has the goal to develop a health service for detecting and preventing frailty among older adults by offering them innovative eHealth services and has a strong basis in their local community. Within this project, we see frailty as the rate of functional decline, whereby people can be robust (no functional decline), prefrail (some to quite some functional decline), and frail (an amount of functional decline that necessitates immediate medical intervention). eHealth interventions for improving physical and cognitive functioning and a website for educating older adults about healthy nutrition were developed in the project for improving older adults' health. The nutritional intervention educates people about healthier choices and to improve food habits, geared towards older adults. The physical intervention focuses on exercising for strength, endurance, and mobility training. The cognitive intervention, finally, targets improving the main cognitive functions: attention, memory, and executive functions. Those older adults that, during a screening, displayed some decline on the nutritional, cognitive, or physical aspect can then make use of these interventions. The service was initially developed for and implemented in two regions: Campania in Italy and Enschede in Netherlands. At the start of the project, an initial, high-level service model was developed by the project partners (including clinicians and experts in telemedicine) that served as input during the initial workshops (which are described below). In this model, the use of technology was foreseen during screening and prevention, but it still remained unclear how older adults could be guided best to the services, what the role of healthcare professionals was, how older adults should be guided during training, and so forth.

### 2.3. Setup in Italy

A first meeting was organized to create awareness among relevant stakeholders about the upcoming service for detecting and preventing functional decline and for the activities which would be deployed within its scope. The meeting (and the workshops that followed) also provided the opportunity to identify existing gaps in terms of digital and health literacy so that support could be arranged for overcoming these gaps and to facilitate the adoption of the innovative, ICT-supported services. A set of organizations that were closest to the older adults in the community (i.e., they are key stakeholders) were invited. These key stakeholders were then expected to discuss the proposed service with others in their vicinity. The stakeholders were selected and contacted based on the knowledge and experience of Caritas. Caritas is an international, religious organization that aims to improve the life and possibilities of people, by working closely with local churches. As in the Campania region (where the service model would at first be deployed), religion and spirituality play a large role in the life of older adults, Caritas and the Catholic Church were taken as the starting point for service design. In the end, 60 people attended the meeting. They were representatives from local hospitals, local nonprofit organizations that were interested in collaborating (such as Caritas or a large, local Judo club), local governments, seniors' associations, and the older adults themselves. During this meeting, the following topics were presented and discussed with all representatives and older adults to elicit the background knowledge needed to set-up local activities for detecting and preventing frailty:

1. the role of older adults within the service and the service design (emphasizing the concept that the older adults are at the same time “object” of the screening, and “partners” in that their feedback was important for optimizing the service design and implementation);
2. the initial service model;
3. a comparison with other, highly-esteemed, sociosanitary services in Italy;
4. making use of voluntary work to support the detection and prevention of functional decline (focusing on health promotion, socialization, and health and ICT literacy);
5. the use of ICT to offer new services and the integration of the new services with existing medical information systems;
6. how to provide older adults with an environment that makes it easier to make a life-altering change towards a healthier lifestyle.

This meeting led to an ideal service model that was subsequently detailed during nine meetings with representatives from two local communities where the service would be made available and the other key-stakeholders, like Caritas, general practitioners, the municipal health service, involved nonprofit organizations, and the local hospital. The goal of these workshops was to transform the ideal service model into a model that could easily be implemented locally by modifying it to fit local contexts in terms of opportunities (available resources), needs, and expectations. An ideal model was used as input for these meetings, as such models can aid a discussion on how to set up a service in a local community [22]. The meetings were held over a period of three months. While the first meeting was plenary (and included an overview of the aims of the service and the ideal service model), in-between meetings were focused more on specific topics (such as developing sessions in which the detection of frailty is combined with health literacy classes for older adults). Over time, the following topics about the anticipated service model were discussed with and among all stakeholders:

1. the current role(s) and task(s) of every stakeholder with regard to detecting, monitoring, and preventing functional decline;
2. the gain of every stakeholder to participate in and use the service;
3. the envisioned role(s) and task(s) of every stakeholder within the service;
4. agreement on the division of role(s) and task(s) among the stakeholders.

### 2.4. Setup in the Netherlands

A first workshop had the goal to create an “ideal” service model. This was done with a wide range of stakeholders and end-user representatives: a community nurse, a specialist in geriatric medicine, a general practitioner, a manager of a homecare organization, a representative from a municipality, a representative from a seniors' association (who acted as end-user representative), and a representative of a municipal health service. These stakeholders were selected after a brainstorm in which as many relevant stakeholders as possible were listed. Subsequently, they were ranked on three attributes that make up stakeholder salience: power, legitimacy, and urgency [23]. This ranking was used to determine which stakeholders were most needed at the workshop. After an introduction, the following subjects were discussed:

1. each participant's current roles and tasks regarding detecting and preventing frailty and functional decline;
2. an inventory of problems with the current way frailty is dealt with in care;
3. introduction of a conceptual service model (e.g., demonstrating the options technology provides for training health on a distance);
4. listing the roles and tasks each participant sees for himself/herself in the new service model;
5. mapping the perfect procedure for a fictitious persona.

This workshop led to an “ideal” service model that was subsequently refined in a set of nine meetings and workshops with representatives from local organizations that resided in the area in which the service would be deployed for the first time. Like we mentioned above, this tactic was used to aid the discussion on how to set up a service in a local community. These nine meetings were held with the municipality, a healthcare center, a healthcare insurer, a physical therapy practice, a healthcare organization for older adults, a community center, the University of Applied Sciences, and the researchers. The goal of these workshops was to transform the ideal service model into one that would fit the stakeholders' and users' contexts and routines. These meetings were also held in a time span of three months. Not all meetings were held with all stakeholders. Where the first and last meeting were plenary, in-between meetings were held to discuss issues affecting mainly a few stakeholders (such as creating a student assignment that allows students of the Applied University to do an internship within the project). The topics that were addressed during these meetings were similar to those in Italy: determining current roles of the stakeholders, assessing their potential gain, determining the role(s) and task(s) each stakeholder would like to have, and reaching consensus over the division of roles and tasks.

### 2.5. Data Analysis

Both plenary meetings in Italy and Netherlands (where the “ideal” service models were created) were audio-recorded and transcribed. Next, they were analyzed by means of thematic analysis whereby stakeholders' and end-users' interests with respect to frailty care, and their preferred role(s) and task(s) within the process of detecting and preventing frailty were mapped. The ideal service model was mapped by means of an activity diagram. In Italy, the subsequent meetings to refine the service model were documented via minutes of the meetings that were confirmed by all parties present. In Netherlands, after each meeting an improved version of the service model was created that was discussed in a subsequent meeting until all stakeholders agreed that this was a suitable service model. The final service models were also mapped by means of activity diagrams.

## 3. Results

### 3.1. The “Ideal” Service Model

The initial meetings that were used to come to an “ideal” service model resulted in two important insights about the way frailty care is currently provided; detection and prevention of frailty is dispersed over many different actors who do not collaborate, and there is currently no shared language for dealing with frailty. These views were shared in both countries. Next, for the to-be-developed service all stakeholders and end-users agreed on the following starting points.

1. The older adult is responsible for the management of his or her own health and should be the party that takes initiative when health problems should be addressed (urgently).
2. General practitioners want to be informed about the results of the screening, and there is a need to integrate their routine activities with the novel services for the detection and prevention of frailty and functional decline. Hereby, the aim is not to provide any additional work for them.

Alongside, in Italy it became apparent that there is a need for activities that educate older adults about working with ICT technology (e.g., the internet). Ideally, activities with regard to frailty detection and prevention should be combined with meetings in which older adults learn to work with ICT.

**Figure 1:**
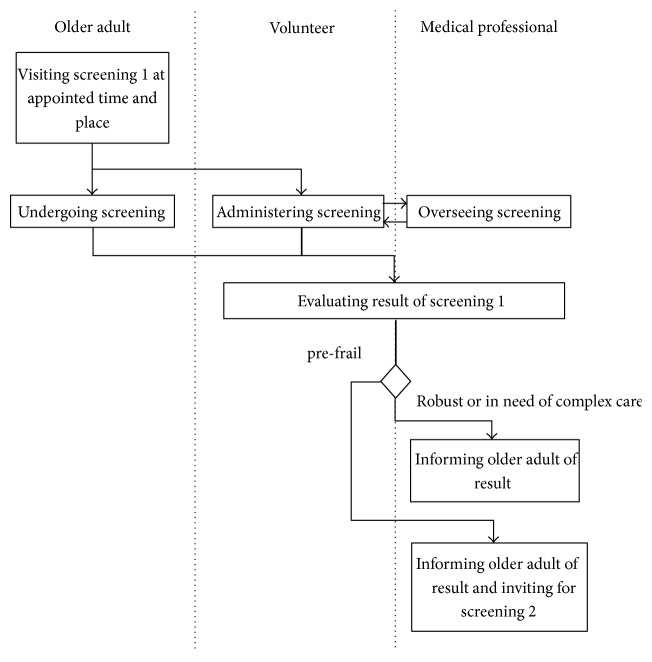
Excerpt of the “ideal” service model activity diagram for Italy.

The ideal service models that were discussed resulted in an initial design in which older adults are invited for a two-stage screening process (one consisting of questionnaires, one consisting of physical and cognitive tests and additional questions) that identifies their rate of functional decline and classifies them as either robust, prefrail, or frail. While frail individuals are referred to a medical professional, and robust people receive an invitation for a new screening a year later, the group of prefrail people (those with some functional decline) are offered eHealth interventions for improving their cognitive functioning and physical functioning or for educating them about healthy nutrition for older adults. These services can be accessed at home, or, for the Italian context: at the local church, where the older adults are helped by trained volunteers. During the process, the general practitioner is kept up to date about the results and is only involved when an older adult needs urgent attention. For the two contexts, the ideal service model was mapped by means of activity diagrams (see Figure 1 for an excerpt in which an older adult is invited for and undergoes a first screening). These activity diagrams then served as input for the subsequent meetings in which the service model was further refined.

### 3.2. Refining the Ideal Service Model

In a series of meetings with local stakeholders in Italy and Netherlands, the ideal service models were refined, so that they could be implemented in practice.

In Italy, the subsequent meetings were used to formalize the division of tasks and to make this official by specific agreements. Next, tasks were divided and new organizations were included as collaborators, like Health Campus, a nonprofit organization that involves more than 200 volunteer clinicians and focuses on providing free health screenings and promotes healthy lifestyles. Finally, the need for solving the existing health and ICT literacy gaps was addressed. The solution was found in an interactive “nutrition meeting,” where people learned about healthy nutrition, were provided essential information on preventive medicine, and were in parallel taught ICT skills (use of office functionalities, to write down recipes, prepare PowerPoint presentations, access, and navigation on the internet to search for nutritional information, etc.). This way, they could gain the knowledge necessary for making well-informed dietary choices, and could receive additional information from professionals on topics they identified as relevant. At the same time, they could also learn the ICT skills that are needed in today's society and for making use of the service's ICT modules. To support the ICT training, another non-profit organization, Progetto Alpha, collaborated. Support was also provided by general practitioners of “Salute in Collina,” by the Campania Regional Center for Urban Veterinary Safety and by the Department of Public Health of Federico II University.

In Netherlands, local general practitioners were doubtful about the feasibility of providing screening and training services via the internet only. They thought many older adults would not have access to the internet. Furthermore, they would like to see older adults leaving their house and mingling with other people, as loneliness is quite a big problem. Therefore, we explored new collaborations for opening up screening and training services in the local community. New partners were happy to be involved and it was agreed upon that kiosks will be opened at a local physical therapy practice, a healthcare organization for older adults, and the local community center. Next, the need for a cost-effective, sustainable business model led to the idea to involve the local University of Applied Sciences, which is always in a need for places for their students to do an internship. The University of Applied Sciences was also willing to collaborate and it was decided upon that their students (physical therapy and nursing) will support the logistics behind the service (e.g., sending out invitations) and will support the older adults during the screening and training, both at home and at a location within the community.

### 3.3. A Community-Based, Technology-Supported Service Model for Detecting and Preventing Frailty among Older Adults

**Figure 2:**
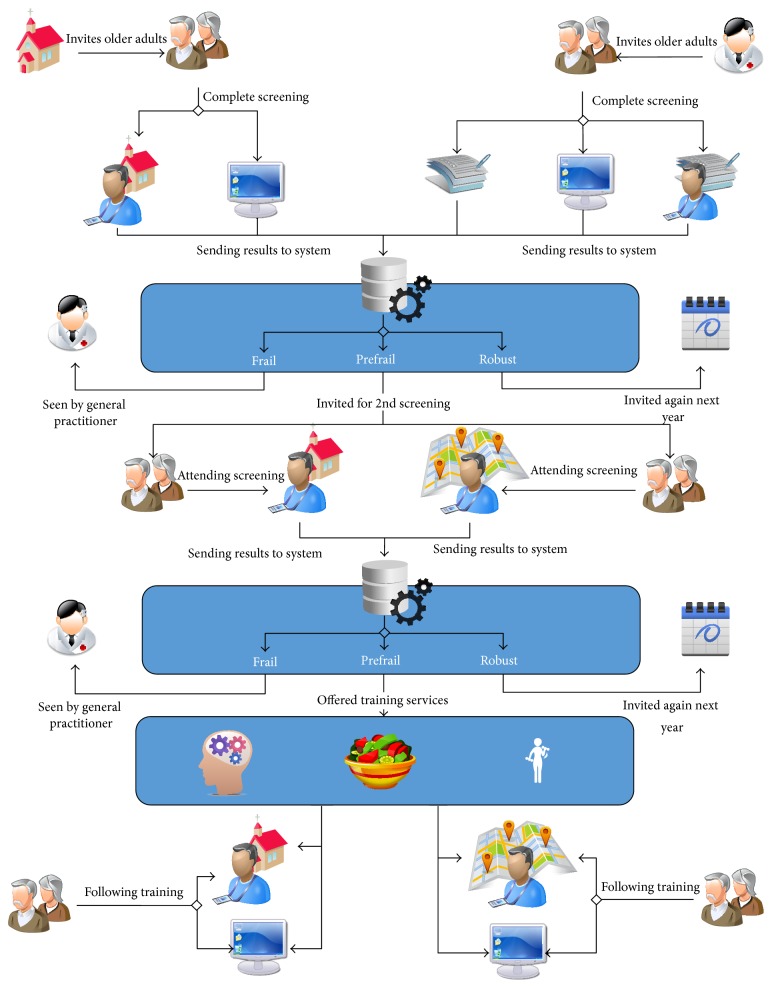
Service model.

**Figure 3:**
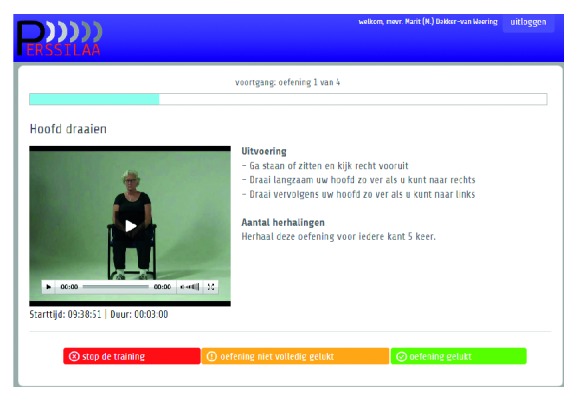
Screenshot of the service for training physical functioning.

Figure 2 displays the service model that was created on the basis of the ideal service models and the alterations that were made in the subsequent meetings. In the service model, there are unique routes for entering the screening and training services. In Italy, older adults are invited for the first screening via the church, after which they can complete this screening at the church with the help of a trained volunteer or online. As older adults in Italy frequently visit the church, where many activities take place, this appeared to be the best way to reach them. In Netherlands, older adults are invited by the general practitioner's office (in name only, logistics are handled by the students) after which they can complete the first screening at home on paper, online, or with the help of a trained student. The general practitioner was chosen as the party sending out the invitations as this would yield the highest response from the older adults, due to his or her authority. This first screening consists of a set of questionnaires to assess an individual's general health status and his or her physical and cognitive status, as well as their nutritional habits (by means of questionnaires like the Groningen Frailty Indicator and the Mini Nutritional Assessment). In all cases, the results of the first screening are sent or uploaded to a central database that also determines the outcome for every individual (using cut-off scores for the different survey instruments that classify an individual as frail, (pre)frail, or robust, based on their rate of functional decline). Frail individuals are referred to a medical specialist (e.g., the general practitioner), who will, after personal examination, make a diagnosis and determine a treatment plan. Robust individuals will receive an invitation for a new screening a year later. An individual is classified as prefrail when limited decline is identified on at least one of the following three domains: nutrition, cognition, or physical status. These prefrail individuals are invited for a second screening. The second screening is administered by a trained volunteer at the church (Italian context) or by a trained student at different locations in the community (Dutch situation). This second screening consists of physical and cognitive tests, as well as some additional questions. Again, results are sent towards the database that, again, classifies every individual. This time, frail people are sent to a medical professional, robust people receive an invitation for a new screening a year later, and prefrail individuals are offered training services (for training physical or cognitive functioning or to educate people about healthy nutrition for older adults). These training services can be used at home via an online service. See Figure 3 for a screenshot of the service for training physical functioning, where people are instructed to do exercises via video, audio, and text. Alternatively, in Italy older adults can make use of the training services at the church and in Netherlands at the same locations in the community at which they could join the screening.

## 4. Discussion

In this paper, we have discussed how we have created a community-based, technology-supported service model for the detection and prevention of frailty among older adults. We applied a participatory design approach to come to a useful model that easily aligns with working procedures and the designated context of work. In the service model, older adults are screened via different modalities (online, on paper, or in person at a location in the community) after which they are provided training services for improving their physical or cognitive functioning, or for educating them about healthy nutrition for older adults. These training services are also offered via different modalities (as online, self-service module or at locations in the community). Medical professionals (such as general practitioners and nurse practitioners) are informed about the medical status of each older adult but do not play an active role unless the situation calls for it.

The service model and the technology can be used as a basis for developing a similar service model in a wide range of regions, countries, or healthcare models. But when one embarks on implementing the service model, several steps need to be localized. The way in which older adults are guided towards the screening and training services needs to be adapted to the traditions, possibilities, and stakeholder needs in a specific region. For example, we found that in Italy, the church is one of the most suitable venues for inviting and helping older adults, the general practitioner should invite people, while trained students at different locations in the community can aid older adults in their training efforts. Other assumptions within the service model (how the General Practitioner should be informed about results, and how to integrate the novel services supporting the screening for frailty to the advantage of his routine activity and especially of his patients) should be checked. Ideally, this should be done by conducting stakeholder meetings in which the key players for a setting in which the service is to be implemented gather and discuss the adaptations that need to be made to the service model we presented. It could take a few meetings before consensus is reached, but the investment in terms of time and effort should be quite small. Specifically, we see the following issues that need to be discussed. First, one must assess how the service model can be streamlined with existing working procedures. One should especially determine which actor is most suitable for inviting older adults for a screening, and which actor(s) should host and support the local screenings and training services in the community. Second, as the different medical professionals in a region will want to be kept up-to-date about the results of the screening and progress of training, connections will need to be made between the online frailty service and database and the local medical information system(s). As it differs per country or even region which medical information systems are used, one will need to determine which system to connect to, how to make this connection, and how and to whom to display results from the screening and training in the specific medical information system. For example, in Netherlands, general practitioners stated that they wanted to receive the results of each screening to be incorporated into a patient's electronic health file as an episode with an ICPC code (for overall decline). Third, a business model should be created that specifies how all the different activities are financed and how all the stakeholders are rewarded for their efforts. Without such a business model, the service will not be sustainable beyond the point where seed money runs out.

The setup of the service model (with a special focus on technology-supported, community-based care) has the potential to be cost-effective. It provides easy access for older adults towards healthcare service (as they can access them online or visit them in their local neighborhood). Finally, due to the use of online tools for screening and training, the service can scale up easily. As a result of these advantages, the service model can be a solution for the challenges that healthcare is currently facing: to provide care to a growing number of older adults with fewer resources, all the while ensuring that people retain a good quality of life. Via this service model, care is moved from a reactive, cure-oriented approach that is centered at medical institutions and medical professionals towards a prevention-focused model that takes advantage from patient empowerment, online services, and trained volunteers, in a health-aware, responsible community. Consequently, the demand on the healthcare system decreases, shifting to prevention and health promotion, while citizens take an active role in their health, are empowered in receiving the care they need in the form they want (online, in the community, or as a combination), and improve their overall health outcomes.

The use of participatory design with stakeholders and end-users has resulted in a service model that not only is innovative but also aligns with the context and working routines in which it is to be implemented. Apart from these benefits, their involvement has, as we have experienced, also other advantages. First, as they become not only consumer of the service but also creator, stakeholders and end-users are more willing to adopt the service model and associated technology. After all, it is also partly “theirs.” Second, for many end-users, adopting a new service (especially one that is fundamentally different from the traditional healthcare services they are familiar with) requires a paradigm shift. In our study, we saw that the older adults need to realize they should not rely on a doctor to arrange everything for them but should seize control themselves. Involving end-users into design provides them with the opportunity to get used to the idea and to discuss it with their peers. Ultimately, this leads to an implementation context where end-users are more willing to accept a new service's starting points.

## 5. Limitations

The development of the service model was done in participation with stakeholders and end-users but focused on a hypothetical situation. Existing initiatives for detecting and preventing frailty are scarce, so all participants had to talk about and imagine a future scenario. It might well be that they missed barriers that hinder successful implementation and service delivery and the only way to identify these barriers is to implement, execute, and evaluate the service. Then, with the insights from this evaluation, the service model can be further improved.

As we mentioned before, the service model cannot be copied “as is” to a new region where working procedures and the implementation context are different. Instead, one should adapt the service model to fit a specific region following the steps we have formulated above.

## 6. Concluding Remarks

The service model that we have developed is unique in that it is the first to detect and prevent frailty by means of community-based, technology-supported services. As such, it marks a step forward in creating healthcare services for older adults that not only include care by professional caregivers to deal with acute problems but also lead to a set of healthcare services that prevent problems by integrating initiatives in the community with online services. Our next steps are to implement the service model in different regions in Italy, in Netherlands, and in EU, taking advantage of the Reference Site Collaborative Network of the European Innovation Partnership on Active and Healthy Ageing (all the while improving the service by taking into account the lessons that the actual deployment provides us) and to evaluate the services in terms of clinical effectiveness and adoption by stakeholders. This way, we can also assess whether the model lives up to its potential.
